# Hyposmia in Parkinson’s disease; exploring selective odour loss

**DOI:** 10.1038/s41531-025-00922-3

**Published:** 2025-04-04

**Authors:** Eleanor Mitchell, Christian Mattjie, Jonathan P. Bestwick, Rodrigo C. Barros, Artur F. Schuh, Cristina Simonet, Alastair J. Noyce

**Affiliations:** 1 Centre for Preventive Neurology, Wolfson Institute of Population Health, Faculty of Medicine and Dentistry, Queen Mary University of London, Charterhouse Square, London, UK; 2 Machine Learning Theory and Applications Lab, School of Technology, Pontifical Catholic University of Rio Grande do Sul, Porto Alegre, Rio Grande do Sul, Brazil; 3 Department of Pharmacology, Universidade Federal do Rio Grande do Sul, Rio Grande do Sul, Brazil; 4 Neurology Department, Hospital de Clínicas de Porto Alegre, Rio Grande do Sul, Brazil; 5 Department of Clinical & Movement Neurosciences, UCL Institute of Neurology, London, UK

**Keywords:** Parkinson's disease, Predictive markers

## Abstract

Smell loss is a frequent and early manifestation of Parkinson’s disease (PD), serving as a sensitive - albeit nonspecific - clinical biomarker^1^. The notion that PD causes odour-selective hyposmia has been debated for three decades. Previous studies have used healthy controls as the comparator; this is problematic given the majority presumably display normal olfactory function. Using University of Pennsylvania Smell Identification Test data from the Parkinson’s Progression Markers Initiative, we trained eight machine learning models to distinguish ‘PD hyposmia’ (*n* = 155) from ‘non-PD hyposmia’ (*n* = 155). The best-performing models were evaluated on an independent validation cohort. While specific responses (e.g. mistaking pizza for bubble gum) were impactful across models, at best only 63% of PD cases were correctly identified. Given we used a balanced data set, 50% accuracy would be achieved by random guessing. This suggests that PD-related hyposmia does not exhibit a unique pattern of odour selectivity distinct from general hyposmia.

## Introduction

Parkinson’s disease (PD) is currently defined by the presence of ‘hallmark’ motor symptoms, classically bradykinesia, rigidity and tremor^2^. However, these features correlate to a relatively advanced level of nigrostriatal degeneration, potentially explaining why neuroprotective trials in those with clinically diagnosed PD have so far yielded disappointing results. There is a growing consensus that early detection will be crucial to the development of disease-modifying therapies, with efforts to identify suitable biomarkers underway^3,4^. Smell loss (hyposmia) is a sensitive clinical biomarker for early PD, however specificity is limited by the numerous common causes of, and diseases associated with, olfactory dysfunction^5,6^.

The notion of a PD-specific, odour selective pattern of hyposmia has been debated for nearly three decades. At least 18 studies^6–21^ have addressed the concept, typically following a similar design. The University of Pennsylvania Smell Identification Test (UPSIT)^6,8,19,22,23^, Sniffin’ Sticks test^7,16–18^ or T&T olfactometer^20,21^ is administered to PD patients and age/sex matched healthy controls; the ability of a subset of odourants to differentiate cases from controls is then compared to the full test. One consistent finding is that tests can be truncated - in the case of the UPSIT to at least 12 of the 40 items - without significantly affecting performance. However, a consensus has never been reached regarding the best-performing odour combination; for a visual summary, see Vaswani et al.^19^. Only two studies^8,11^ have suggested that certain subsets might actually *outperform* the full UPSIT. Bohnen et al.^10^ also reported that the inability to identify banana, liquorice and dill pickle was more indicative of nigrostriatal denervation on DAT scanning than the total 40-item score.

Reviewing the research to date, a lack of reproducibility is notable. In 2018, Morley et al.^6^ re-examined three previously proposed subsets^8,10,23^ in over 300 cases and controls. All odour combinations demonstrated a substantially lower sensitivity and/or specificity than originally reported. Moreover, Vaswani et al.^19^ evaluated 16 abbreviated UPSITs supported by existing literature, noting no single UPSIT item featured in >50% of these subsets. Indeed, all but three of 40 items appeared in at least one study.

There are several possible explanations for this, an obvious one being that there is no odour selectivity in PD^22^. However, other factors should be considered:

(i) **Healthy controls have (almost) always been the comparator**, of which the majority presumably display normal olfactory function. Likewise, at least 10% of a PD cohort is typically normosmic^24^. To draw robust inferences, cases of PD-related hyposmia should be compared with other causes of hyposmia. This design was implemented in one small study^17^, concluding little evidence for PD-specific odour-selectivity.
(ii) **Samples have been small and heterogenous**. With recent exception - including two analyses by our group^13,15^ - studies have been statistically underpowered. For the 18 aforementioned papers, the median number of cases and controls were 67 and 75. Studies were based in the USA^6,10,11,14,19,22^, UK^6,9,12,13,15,23^, Germany^7,16,17^, Australia^8^, Austria^18^, Italy^18^, The Netherlands^18^, China^20^ and Japan^21^. Regional differences in familiarity with odourants are inevitable.
(iii) **Different smell tests were used**. Most studies used the US UPSIT^6,14,19,22,23^; one assessed a 12-item adaptation^8^. Two studies examined items common to the US/UK UPSITs^13,15^. The remainder used the Sniffin’ Sticks test^7,16–18^ or the T&T olfactometer^20,21^.
(iv) **Varying degrees of methodological rigour were applied**, from descriptive statistics to more complex data-driven approaches analysing millions of odour combinations^12,15^.

Overinterpretation of findings from individual studies is a tangible risk. However, odour selectivity remains an avenue worth exploring. If it exists, the development of PD-specific smell tests could improve the positive predictive value, convenience and cost of early detection methods^24^.

The availability of large, open-source datasets, combined with the development and wider application of machine learning (ML) allows for new approaches to a 30-year-old question. Using data from the Parkinson’s Progression Markers Initiative (PPMI), we compared 194 hyposmic PD participants with an age/sex matched cohort of 194 hyposmics without PD. We determined whether ML models could identify PD by analysing UPSIT response patterns.

## Results

### Participant selection

**Fig. 1:**
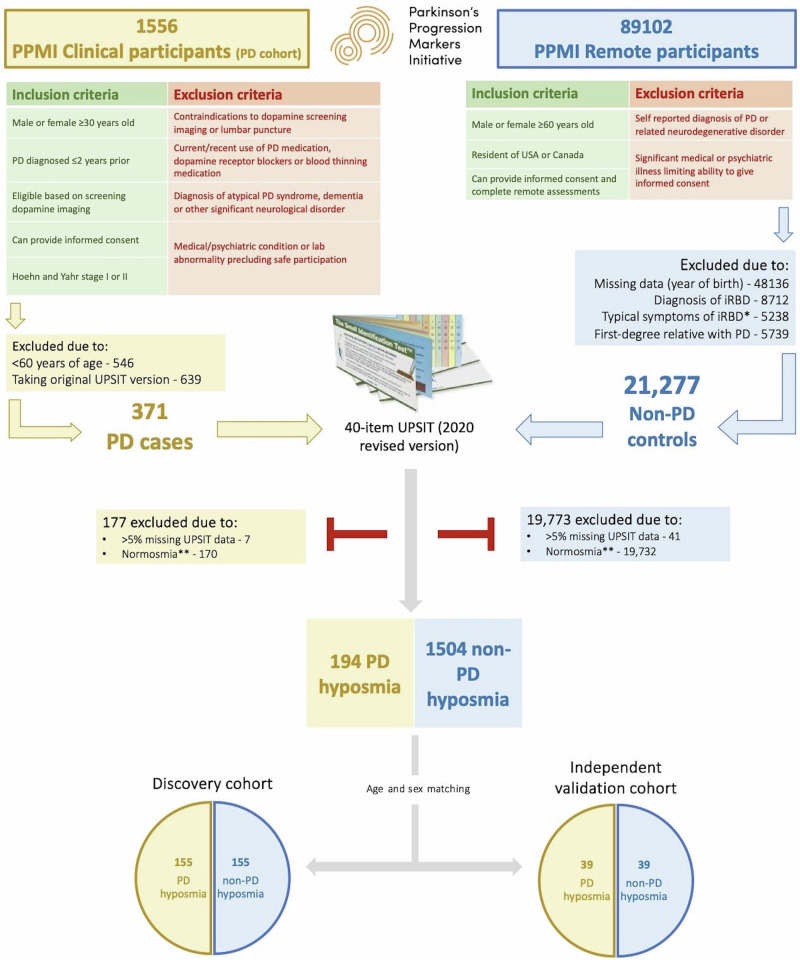
Overview of participant selection. PPMI Parkinson’s Progression Markers Initiative, PD Parkinson’s disease, UPSIT University of Pennsylvania Smell Identification Test. *Acting out dreams, punching, kicking, yelling or falling out of bed; **UPSIT score ≥15^th^ percentile^38^.

Figure 1 outlines the hyposmic participant selection process. After excluding normosmic PPMI participants (170 PD, 19,732 non-PD), or those with >5% missing UPSIT responses (7 PD, 41 non-PD), 194 participants with ‘PD hyposmia’ remained. We then randomly selected 194 age and sex matched participants with ‘non-PD hyposmia’ from a total pool of 1504.

### Cohort comparisons

**Fig. 2:**
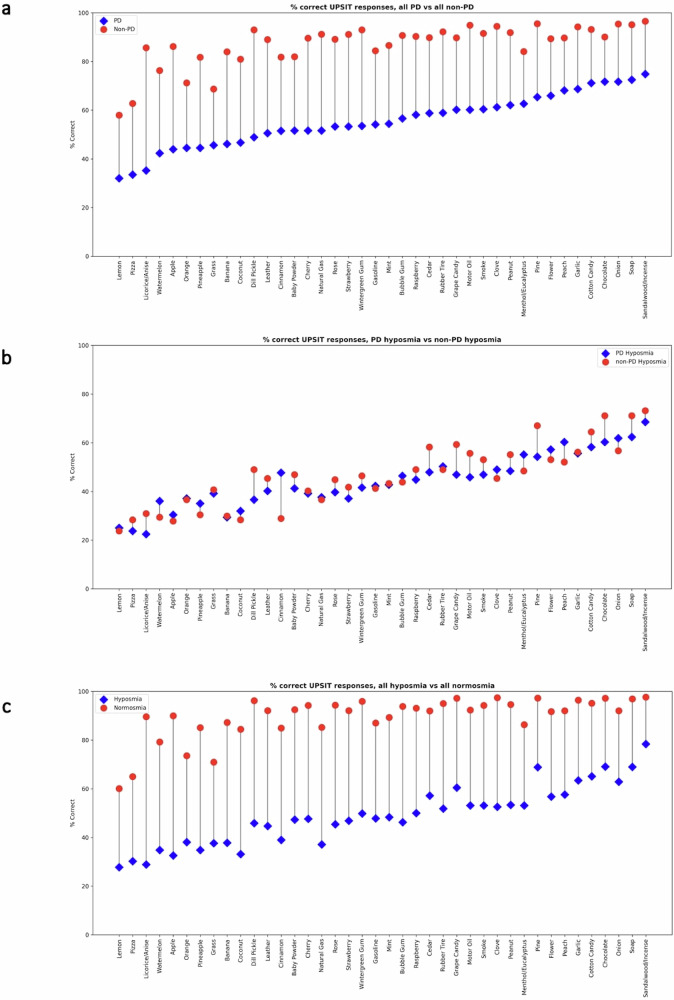
Percentage of participants answering correctly for each UPSIT item, within three different cohort comparisons. **a** All PD (*n* = 364) vs. all non-PD (*n* = 21,236), **b** PD hyposmia (*n* = 194) vs non-PD hyposmia (*n* = 194), **c** All hyposmia (*n* = 1698) vs. all normosmia (*n* = 19,902). PD Parkinson’s disease, UPSIT University of Pennsylvania Smell Identification Test.

Figure 2 shows the percentage of participants answering each UPSIT item correctly, across three cohort comparisons. First, all participants with PD (*n* = 364) were compared to those without PD (*n* = 21,236), regardless of hyposmia status. Then, participants with ‘PD hyposmia’ (*n* = 194) were compared to an equal number of participants with ‘non-PD hyposmia’. Finally, PD status was disregarded and participants were classified simply as hyposmic or normosmic (*n* = 1698 and *n* = 19,902, respectively). Unsurprisingly, participants with PD had lower UPSIT scores than those without PD (Fig. 2a). However, when the comparison was limited to hyposmic participants (Fig. 2b), the distribution of correct answers was similar. Indeed, when ignoring PD status and comparing hyposmia with normosmia (Fig. 2c), the resultant graph resembled Fig. 2a. This implies correct UPSIT responses are comparable between PD-related and all-cause hyposmia.

### Baseline characteristics of hyposmic participants

Hyposmic participants were predominantly male (67%) and white (93%). Those with ‘PD hyposmia’ had an average disease duration of <1 year. See Table 1 for a summary of participant characteristics.

**Table 1:** Baseline characteristics of study participants

Characteristic	PD hyposmia *n* = 194	Non-PD hyposmia *n* = 194
Age, mean ± SD	68.04 ± 5.69	68.13 ± 5.64
Male sex, *n* (%)	130 (67)	130 (67)
White ethnicity, *n* (%)	180 (93)	182 (94)
PD duration (years), mean ± SD	0.94 ± 0.58	–

*PD* Parkinson’s disease, *SD* Standard deviation.

### PD-hyposmia vs non-PD hyposmia

Chi-squared tests revealed that correct identification rates for pine, cinnamon, soap, dill pickle, grape candy, chocolate and licorice/anise were significantly different (*p* < 0.05) between ‘PD hyposmia’ and ‘non-PD hyposmia’. However, when adjusting for multiple comparisons, only pine and cinnamon retained significance (see Supplementary Table 1).

### Discovery cohort

We then trained ML models to predict ‘PD hyposmia’ on the basis of their UPSIT responses. The mean performance of the 10 best-performing ML approaches in the discovery cohort are shown in Table 2 (for full results, see Supplementary Table 2). Considering our data set featured an equal number of participants with and without PD, 50% accuracy would be achieved by random guessing alone. As such, none of the models performed particularly well, with the highest accuracy at 68%. The inclusion of specific UPSIT responses (e.g. mistaking cinnamon for tomato) improved performance, albeit to a modest degree (see Supplementary Table 2). This is noteworthy given previous studies have predominantly characterised UPSIT responses as correct or incorrect (i.e. have not accounted for which incorrect answer was selected). Inclusion of age and sex did not increase accuracy; indeed, two of the four best-performing models omitted demographic features. We then excluded PD participants with missing UPSIT data (*n* = 69; an equal number of matched non-PD participants were removed to retain a balanced dataset). Results did not substantially change (see Supplementary Table 3).

**Table 2:** Performance analysis of 10 best-performing ML approaches, predicting PD based on UPSIT responses

Feature set	ML model	Accuracy	Specificity	Sensitivity	Precision	F1-score
Response_SA	XGBoost	0.68	0.70	0.82	0.69	0.68
Response_SA	Gradient Boost	0.68	0.72	0.80	0.69	0.66
Response	Gradient Boost	0.68	0.74	0.79	0.70	0.66
Response	XGBoost	0.67	0.68	0.82	0.68	0.67
Response	Extra Trees	0.64	0.61	0.81	0.63	0.64
Response_SA	Extra Trees	0.63	0.63	0.80	0.63	0.63
Response	SVM	0.63	0.61	0.80	0.63	0.63
Response_SA	SVM	0.62	0.61	0.80	0.62	0.62
Response	Random Forest	0.61	0.59	0.80	0.61	0.62
Response_SA	Random Forest	0.61	0.61	0.78	0.61	0.61

Models were trained on a discovery cohort of hyposmic participants (194 PD hyposmia, 194 non-PD hyposmia) using four different combinations of features. Metrics were computed using leave-one-out cross-validation. For full results, see Supplementary Table 2.

*ML* Machine Learning, *SA* inclusion of sex and age in the model, *SVM* Support Vector Machine, *UPSIT* University of Pennsylvania Smell Identification Test.

### Independent validation cohort

The four best-performing approaches were applied to the independent validation cohort (see Table 3). As expected, accuracy was universally reduced when models were presented with new data. Nevertheless, the models performed better than random guessing, indicating that there might be subtle differences between PD- and non-PD hyposmia. The highest performing approaches use multiple decision trees for their prediction; for two illustrative examples, see Supplementary Fig. 1.

**Table 3:** Performance of the four best-performing approaches in the discovery cohort, using all UPSIT response features, with and without age and sex features, when applied to an independent validation cohort of 39 ‘PD hyposmia’ and 39 ‘non-PD hyposmia’

Feature set	ML model	Accuracy	Specificity	Sensitivity	Precision	F1-score
Response	Gradient Boost	0.63	0.56	0.83	0.61	0.65
Response_SA	Gradient Boost	0.63	0.59	0.82	0.62	0.64
Response	XGBoost	0.60	0.59	0.78	0.60	0.61
Response_SA	XGBoost	0.60	0.56	0.80	0.60	0.62

*ML* Machine Learning, *SA* inclusion of sex and age in the model, *UPSIT* University of Pennsylvania Smell Identification Test.

### SHAP analysis

**Fig. 3:**
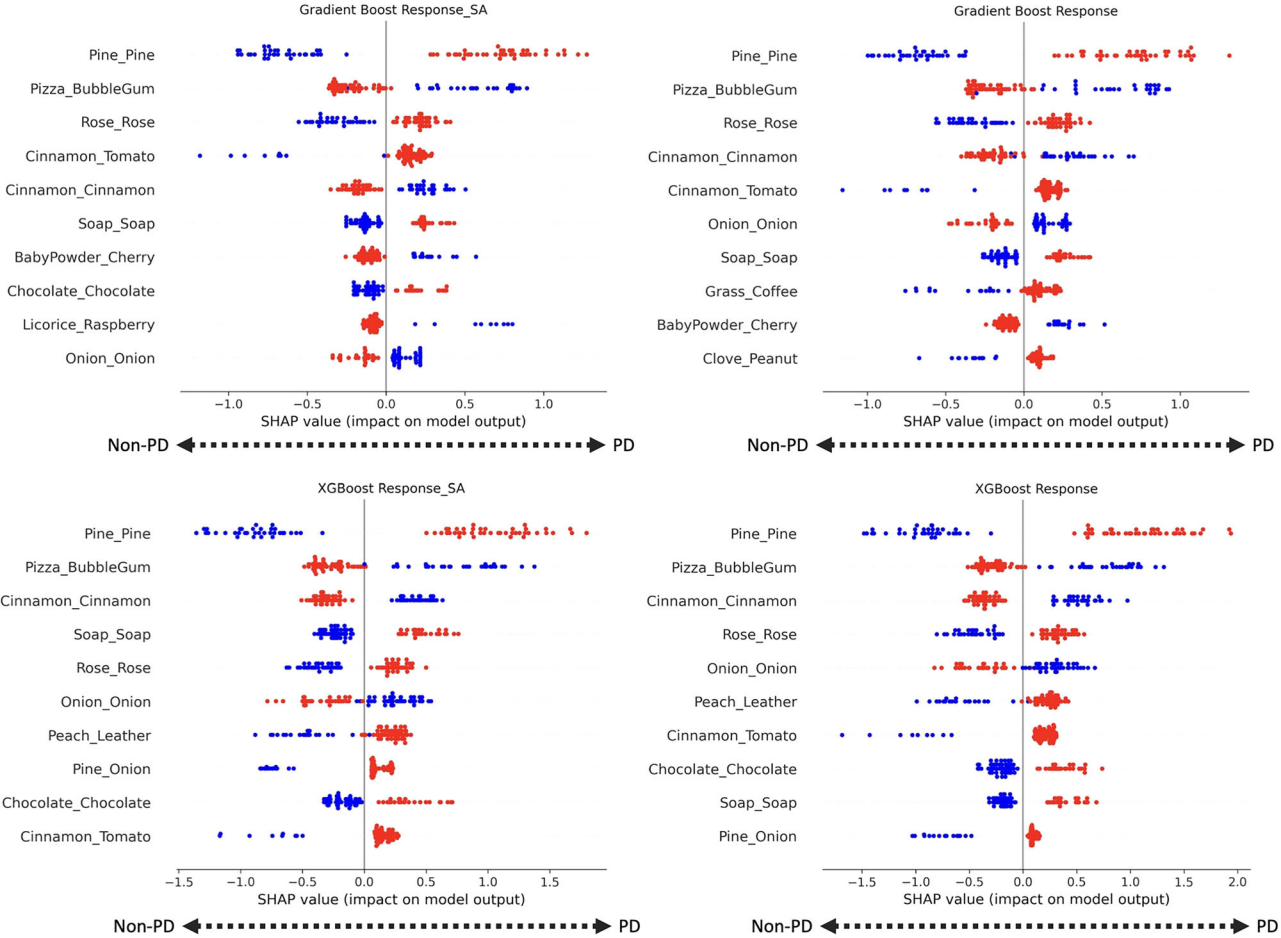
SHAP summary plots for the four highest-performing ML models distinguishing ‘PD hyposmia’ from ‘non-PD hyposmia’. The y-axis lists specific UPSIT responses in the format *correct answer_given response*. Each dot represents an individual participant’s response. Red and blue denote negative and positive responses, respectively; e.g. for *Pine_Pine*: a blue dot indicates that Pine was correctly identified, while a red dot indicates any other response. For *Pizza_Bubblegum*: a blue dot indicates that Pizza was mistakenly identified as Bubblegum, while a red dot indicates any other response. The x-axis represents the SHAP value, a measure of impact on the model’s prediction. Higher SHAP values (toward the right) are associated with PD, whereas lower SHAP values (toward the left) are associated with non-PD.

To further investigate, we examined the importance of particular UPSIT responses using SHapley Additive exPlanations (SHAP), a framework widely used to explain ML model predictions^25^. Figure 3 shows feature summary plots for the four highest-performing models. Certain UPSIT responses were impactful across models. Most notable were the correct identification of pine (correlated with non-PD hyposmia) and cinnamon (correlated with PD hyposmia). Interestingly, the confusion of pizza for bubble gum was also associated with underlying PD. This suggests that prediction can be enhanced by examining *how* rather than just *whether* an item is answered incorrectly. Incorporating age and sex did not impact model performance (see Table 3) and, correspondingly, these features were not relevant in the two approaches that included them.

### PD vs non-PD

Finally, we repeated our analysis, this time disregarding hyposmia status during participant selection. Instead, 364 PD participants were compared to 364 age and sex matched non-PD participants in a discovery cohort (291 PD, 291 non-PD) and independent validation cohort (73 PD, 73 non-PD). The best-performing model could differentiate PD from non-PD with 86% accuracy in both the discovery and independent validation cohort (see Supplementary Tables 4 and 5). However, when applying a simple cut-off method that classified participants with an UPSIT score <30 as presumed PD, a matching 86% accuracy was achieved. This suggests our ML models are largely basing predictions on overall scores, not specific response patterns.

## Discussion

Our analyses indicate that PD does not uniquely affect the perception of specific odours compared to other causes of hyposmia. The three best-performing models were able to distinguish ‘PD hyposmia’ from ‘non-PD hyposmia’ only 63% of the time. Since the test set was balanced, a success rate of 50% would be expected by random guessing. While certain UPSIT responses—such as mistaking cinnamon for tomato—contributed more significantly to these models, this result must be interpreted in light of the models’ overall poor performance. It is certainly possible that particular UPSIT items have greater discriminatory power than others *in the detection of hyposmia*. After all, humans are more sensitive to (and familiar with) certain smells than others. However, the identification of general hyposmia should not be conflated with the early detection of PD.

Hyposmia is one of the most common features of PD. The prevalence of hyposmia is approximately 90% in manifest disease; substantially higher than tremor, for example^1,19,26^. A 2019 meta-analysis found that hyposmia is associated with a 3.84-fold increase in lifetime PD risk^27^. For hyposmic individuals with an affected first-degree relative, the lifetime risk of PD may be as high as 10%^28,29^. Crucially, hyposmia can precede the onset of motor symptoms by at least 4 years, but then appears to remain stable throughout the disease^19,24,28,30^ and is associated with abnormal α-synuclein aggregation in the cerebrospinal fluid^31^. The presence of hyposmia can reliably differentiate PD from atypical forms of parkinsonism^1,18^. Moreover, validated smell tests are relatively inexpensive and easy to administer remotely.

Nevertheless, the predictive value of hyposmia is diminished by its overall frequency in the general population^28^. In 2017, the prevalence of all-cause hyposmia (defined as a failure to identify at least six of eight common odours) in American adults was ~20%, rising significantly past middle age^32^. By contrast, lifetime risk of PD is about 1–2%^24^. Most often, olfactory deficits arise from normal aging, sinonasal disease, head injury, Alzheimer’s disease or epilepsy^1^. In short, whilst most people with PD have hyposmia, most people with hyposmia will never develop PD. Plausibly, the identification of PD-specific odour subsets could enhance risk prediction tools and ultimately drive recruitment into prodromal clinical trials.

Our models displayed a modest superiority over random guessing; one interesting finding is that certain incorrect responses (such as mistaking pizza for bubble gum) were indicative of PD. Although the most impactful feature for model prediction was identifying pine, all models improved when factoring in specific responses, indicating that mistaking pizza for bubble gum should be at least as - or even more - relevant for predicting PD. This kind of detailed analysis warrants repetition in larger datasets. Nevertheless, the SHAP analysis only highlights the responses used by ML models to make a prediction^25^. Given their overall poor performance, these features may not be relevant at all.

The idea that PD might uniquely diminish the perception of certain odours is attractive; but a biological explanation is lacking. It is not the case that one olfactory neuron detects a single identifiable smell. Olfactory neurons are formed of an encapsulated bundle of approximately 200 olfactory receptor cell (ORC) axons^1^. In total, over six million ORCs project ciliated dendrites into the nasal epithelium, each hosting one of 400 olfactory receptor types. Individual receptors respond to a range of chemical stimuli^1^. Likewise, a given stimuli activates a network of receptors, producing ‘chemical signature’^32–34^. Common recognisable odours are a composite of chemical compounds. Clinical smell testing therefore applies blunt instrument to an exquisitely complex system of detection, spatial mapping and recognition.

PD-specific hyposmia would presumably require some kind of selective injury to the olfactory system, or its regenerative capacity. This is hard to elucidate given the pathogenesis of PD-related hyposmia is largely unknown^5^. Olfaction is mediated by the olfactory and trigeminal nervous system, with the former being particularly impaired in PD^1,33^. Pathogenic α-synuclein aggregation is present early in the olfactory bulbs before it is found in higher brain regions^33,34^. The olfactory epithelium therefore provides a potential gateway through which the disease process is propagated, effectively evading the blood-brain-barrier^1^. Indeed, contact between olfactory receptor cells and the external environment raises the possibility of an environmental trigger (e.g. infection, toxin, or trauma) as the initiating agent^33^. Nevertheless, the exact role of α-synuclein aggregation in olfactory loss remains unclear^1,30^. For example, dopaminergic cell expression is actually increased within the olfactory bulbs of PD patients, despite the early emergence of misfolded α-synuclein in this region^28^.

Moreover, while dopamine plays an important modulatory role throughout the olfactory system^1,33^, PD pharmacotherapy has no discernible impact on smell loss^1,35^. Hyposmia is correlated to a positive α-synuclein seed amplification assay (SAA)^31^ and dopamine transporter deficits on PET/SPECT imaging, but the relationship to disease progression is uncertain^28,36^. Alterations to cholinergic, serotonergic and noradrenergic signalling have been proposed as a common pathological substrate for several neurodegenerative disorders featuring early hyposmia^1,33^. However, available evidence is mostly derived from animal and post-mortem studies. Interpretation of such findings is further complicated by the high degree of interaction between these neurotransmitter circuits^30^.

The lack of definitive answer to the question of odour selectivity is partly related to the limitations of clinical smell testing. Various methods have been developed since the 1980s^1^. Common tests (like the UPSIT) ask participants to identify an odour, usually from a forced multiple choice. Though practical, they provide no information regarding the severity of a specific olfactory deficit. Other more nuanced approaches assess an individual’s ability to differentiate two similar odours, the minimum chemical concentration required for odour detection or measuring the time to habituation upon repeated exposure^33^. Regardless, each method – identification, discrimination, detection and habituation – involves higher cognitive processing. All tests place varying demands on memory, in conjunction with education and cultural background^5,28^.

Although our experiments suggest that PD-related hyposmia does not exhibit an odour selectivity distinct from general hyposmia, smell testing remains valuable to PD research. Abbreviated UPSITs offer a simple and cost-effective way to identify hyposmic individuals, who can then undergo more targeted, PD-specific investigations. As such, when screening for early PD, smell-testing likely offers a useful initial filtering method. Our group has previously shown that abbreviated subsets perform with comparable sensitivity to the full 40-item UPSIT^12,13,15^. Use of shortened subsets can lead to cost savings of approximately >75% against the full UPSIT^15^. An optimised protocol might therefore involve i) large-scale administration of a cheap and minimally burdensome smell test^13^ followed by ii) targeted testing for a second biomarker (e.g. SAA and/or imaging^24^). The first step serves to increase the positive predictive value of the second. Sensitivity can be maximised by incorporating data from both steps into an overall risk score^5,37^. Furthermore, if ML models can offer even modest improvements to accuracy, this might have value when scaled to large populations.

Misclassification will have undoubtedly influenced our study. Our ‘non-PD’ participants were classified by self-report. Although we excluded those reporting iRBD features or a positive family history of PD, our control group probably included some prodromal cases. We expect this number to be small, given the PPMI Remote population is relatively unselected and hyposmia is common. PPMI Remote did not involve α-synuclein seed amplification assay testing, but it would be valuable to repeat our analysis using a control group stratified by SAA status. For PPMI Clinical, PD is classified by consensus, following an expert panel review of clinical, genetic and neuroimaging data. Nevertheless, misdiagnosis is a well-established problem in PD research. In fact, Gerkin et al.^11^ reported that PD cases for whom diagnosis was confirmed post-mortem demonstrate a higher degree of odour-selective hyposmia than those diagnosed clinically.

Additionally, our hyposmia cut-offs were based on normative data from PPMI and the Parkinson Associated Risk Syndrome (PARS) study^38^. Participants were overwhelmingly white (98%) and disproportionately likely to report a first-degree relative with PD (34%). 95% of PARS participants were non-smokers (not recorded for PPMI). The analysis also included those with iRBD and constipation. PD risk is increased in all of these groups. Derived threshold values for hyposmia might therefore be overly stringent if this supposedly ‘normative’ dataset featured a high proportion of prodromal cases. Furthermore, results were derived from the original UPSIT; our study used the revised version. A recent comparison demonstrated that people consistently score lower on the former^38^. True hyposmics might have therefore been unnecessarily excluded from our study. However, we lacked a suitable alternative as threshold values have not been published for the revised UPSIT. Previous evaluations of the original UPSIT included <100 participants in each age bracket over 50 years^39^.

A longitudinal analysis is warranted, but beyond the scope of this study. In PD, olfactory function declines in the pre-diagnostic phase, before reaching an early plateau^40,41^. Accordingly, the PPMI study administers only a single UPSIT to the PD cohort at baseline; repeat assessments are considered unnecessary. For our study, limiting PD participants to those diagnosed within two years also ensured a relatively homogeneous cohort of participants, with minimal confounding from disease progression or treatment effects. However, it is noteworthy that a previous study twice administered a 12-item subset to 14 PD participants, one year apart. There was little consistency in the odours correctly identified by a given individual^42^. It would therefore be valuable to administer UPSITs to a larger group of hyposmic PD participants at annual intervals and determine whether the same specific odours remain impaired.

## Methods

### Hyposmia definition

The UPSIT is a widely used, internationally recognised smell test. It comprises 40 “scratch-and-sniff” microencapsulated odourant strips, each of which participants must identify from a forced multiple-choice of four^43^. We included males and females ≥60 years old who scored poorly on the 2020 revised UPSIT. Hyposmia was characterised as an UPSIT score ≤15^th^ percentile, based age/sex specific threshold values; this is the definition currently utilised in PPMI^38^. Where ≤5% UPSIT responses were absent, we imputed the missing values using the K-Nearest Neighbours method, with four neighbours.

### PPMI clinical

Participants with ‘PD hyposmia’ were sourced from PPMI Clinical (NCT04477785), an ongoing longitudinal, observational, multi-centre study investigating clinical, biological and neuroimaging biomarkers of PD. Protocol information for The Parkinson’s Progression Markers Initiative (PPMI) Clinical - Establishing a Deeply Phenotyped PD Cohort AM 3.2. can be found on protocols.io or by following this link: 10.17504/protocols.io.n92ldmw6ol5b/v2. Briefly, participants with PD are recruited from movement disorder clinics at multiple international sites (97% USA-based^38^). Participants must have a recent diagnosis (≤2 years) and lack of pharmacological PD treatment at baseline (see Fig. 1 for full criteria). Participants undergo extensive baseline assessment, including an UPSIT.

### PPMI remote

Participants with ‘non-PD hyposmia’ were sourced from the PPMI Remote Data Collection Study, a population-wide screening initiative to identify people at increased risk of PD. The study was advertised through various means, (traditional & social media, commercials, charity mailing lists etc.). All channels published a link to https://mysmelltest.org, where participants confirmed eligibility (≥60 years old, without PD, USA/Canada resident) and answered six ‘High Interest Questions’ (see Table 4) before completing an UPSIT at home. We excluded those answering ‘Yes/Not sure/Prefer not to answer’ when asked to report features of iRBD, or a family history of PD.

**Table 4:** Six ‘High Interest Questions’ asked during recruitment into the PPMI Remote study

PPMI remote screening ‘High Interest Questions’	
**1**	Has a health provider told you that you have a sleep problem called REM sleep behaviour disorder?
**2**	Some people act out dreams while they sleep. They may punch, kick, yell or even fall out of bed. Have you been told you act out your dreams? Or do you suspect you may do this?
**3**	Do you have any problems with your sense of smell?
**4**	Do you have a first-degree relative (father, mother, full sibling, child) who has/had Parkinson’s disease?
**5**	Do you think your memory is poor, or even very poor, compared to your peers?
**6**	Have you noticed that you are having more problems with thinking, such as difficulty with memory or concentration, that is a change from your normal abilities?

For the purposes of our study, we excluded those answering ‘Yes/Not sure/Prefer not to answer’ to questions 1, 2 or 4.

A total of 194 PPMI Clinical participants met the inclusion/exclusion criteria for ‘PD hyposmia’. Using the K-Nearest Neighbours method, we randomly selected 194 age and sex matched participants with ‘non-PD hyposmia’ from the PPMI Remote study population.

### Item analysis

Using Chi-squared tests, we compared correct identification rates between ‘PD hyposmia’ and ‘non-PD hyposmia’, per individual UPSIT item. Calculated *p*-values were subsequently adjusted for multiple comparisons using the Benjamini-Hochberg method. *P*-values < 0.05 were considered statistically significant.

### Pre-processing and feature sets

Each group of 194 was split using a 4:1 ratio into a discovery cohort (*n* = 155) and independent validation cohort (*n* = 39). We utilised all 40 UPSIT items containing both correct and response features. Correct features indicate whether the participant correctly identified an odour, whereas response features specify the participant’s actual response. We also investigated including sex and age (SA) into the models, resulting in four feature sets: Correct, Response, Correct_SA and Reponse_SA.

### Machine learning and model validation

Traditional cut-off methods do not leverage the full complexity of UPSIT data and may miss subtle PD-specific response patterns (e.g. mistaking cinnamon for pine). As such, we employed eight machine learning approaches, each with different assumptions and implications: Ridge Regression^44^, Random Forest^45^, XGBoost^46^, Support Vector^47^, K-Nearest Neighbours^48^, Decision Tree^49^, Gradient Boosting^50^, and Extra Trees classifiers^51^. We performed leave-one-out cross-validation for each model to evaluate performance in the discovery cohort. We then selected the best approaches – from 32 possible combinations of feature sets and ML models - for final application in the independent validation cohort. This procedure ensured that our models were validated on multiple subsets of the data, providing a robust assessment of their generalisability to new, unseen data.

We assessed model performance using accuracy, specificity, sensitivity (recall), precision, and F1 score. The importance of specific UPSIT responses to model performance was explored using the SHapley Additive exPlanations (SHAP) framework^25^. SHAP uses a game theory-based approach to assess model performance by training and evaluating the model multiple times, each time including, excluding, or altering a specific feature. This measures the feature’s impact on the model’s performance. The resulting SHAP values and plots illustrate the effect of the feature’s actual values on the model’s predictions for unseen data. All data analysis was performed using Python 3.9.

### Consent and ethical approval

All PPMI Clinical & Remote participants provided written informed consent to participate in the study. The PPMI study is conducted in accordance with the Declaration of Helsinki and the Good Clinical Practice guidelines, following approval of the local ethics committees of the participating sites. The current PPMI Clinical study protocol (#002) received initial WCG approval (IRB Tracking #20200597) on April 20, 2020. The previous PPMI Clinical protocol (#001) received initial IRB approval on May 7, 2010, by the University of Rochester Research Subjects Review Board (RSRB #00031629) and was closed by the RSRB on March 9, 2021. Any questions pertaining to study compliance can be directed to The PPMI Data and Publications Committee (DPC): ppmi.publications@indd.org.

### Reporting summary

Further information on research design is available in the Nature Research Reporting Summary linked to this article.

## Supplementary information

Supplementary Data

Reporting Summary

## Data Availability

Data used in the preparation of this article were obtained on 2024-06-15 from the Parkinson’s Progression Markers Initiative (PPMI) database (https://www.ppmi-info.org/access-data-specimens/download-data), RRID:SCR_006431. For up-to-date information on the study, visit http://www.ppmi-info.org.

## Abbreviations

IDA: Image & Data Archive
iRBD: Isolated rapid eye movement sleep behaviour disorder
KNN: K-Nearest Neighbours
ML: Machine learning
LONI: Laboratory of Neuro Imaging
ORC: Olfactory receptor cell
PD: Parkinson’s disease
PPMI: Parkinson’s Progression Markers Initiative
SA: Sex and age
SAA: α-synuclein seed amplification assay
SHAP: SHapley Additive exPlanations
SVM: Support Vector Machine
UPSIT: University of Pennsylvania Smell Identification Test

## Code availability

The underlying code for this study is available in GitHub and can be accessed via this link https://github.com/cmattjie/UPSIT-PD-Hyposmia/.
