# Supplementary material for: Hyposmia in Parkinson’s disease; exploring selective odour loss
Source: NPJ Parkinsons Dis. 2025 Apr 4;11:67. doi: 10.1038/s41531-025-00922-3 (PMC11971265; doi:10.1038/s41531-025-00922-3)
Supplement: Supplementary file 1 — Supplementary Data [file 41531_2025_922_MOESM1_ESM.pdf]

Supplementary data

UPSIT item	p-value	'PD hyposmia'		'Non-PD hyposmia'		Adjusted p-value
		Mean	SD	Mean	SD	
Pine	0.0000	0.54	0.47	0.67	0.47	<0.0001
Cinnamon	0.0004	0.48	0.50	0.29	0.45	0.0087
Soap	0.0055	0.62	0.47	0.71	0.45	0.0735
Dill Pickle	0.0183	0.37	0.48	0.49	0.50	0.1544
Grape Candy	0.0193	0.47	0.50	0.59	0.49	0.1544
Chocolate	0.0324	0.60	0.49	0.71	0.45	0.2161
Licorice/Anise	0.0487	0.22	0.41	0.31	0.46	0.2663
Cedar	0.0533	0.48	0.50	0.58	0.49	0.2663
Motor Oil	0.0676	0.46	0.50	0.56	0.50	0.3003
Peach	0.1248	0.60	0.49	0.52	0.50	0.4993
Rubber Tire	0.1893	0.50	0.49	0.49	0.50	0.6365
Watermelon	0.1942	0.36	0.48	0.29	0.46	0.6365
Menthol/Eucalyptus	0.2228	0.55	0.50	0.48	0.50	0.6365
Peanut	0.2228	0.48	0.50	0.55	0.50	0.6365
Cotton Candy	0.2515	0.58	0.49	0.64	0.48	0.6556
Smoke	0.2640	0.47	0.50	0.53	0.50	0.6556
Baby Powder	0.3065	0.41	0.49	0.47	0.50	0.6556
Onion	0.3524	0.62	0.49	0.57	0.50	0.6556
Pizza	0.3547	0.24	0.43	0.28	0.45	0.6556
Rose	0.3550	0.40	0.49	0.45	0.50	0.6556
Leather	0.3558	0.40	0.49	0.45	0.50	0.6556
Sandalwood/Incense	0.3714	0.69	0.47	0.73	0.44	0.6556
Pineapple	0.3867	0.35	0.48	0.30	0.46	0.6556
Strawberry	0.4059	0.37	0.48	0.42	0.49	0.6556
Wintergreen Gum	0.4098	0.42	0.49	0.46	0.50	0.6556
Flower	0.4749	0.57	0.50	0.53	0.50	0.7058
Raspberry	0.4764	0.45	0.50	0.49	0.50	0.7058
Coconut	0.5069	0.32	0.47	0.28	0.45	0.7241
Clove	0.5417	0.49	0.50	0.45	0.50	0.7472
Lemon	0.5847	0.25	0.43	0.24	0.43	0.7796
Apple	0.6549	0.30	0.46	0.28	0.45	0.8450
Bubble Gum	0.6832	0.46	0.50	0.44	0.50	0.8540
Grass	0.8358	0.39	0.49	0.41	0.49	1.0000
Natural Gas	0.9163	0.38	0.49	0.37	0.48	1.0000
Cherry	0.9174	0.39	0.49	0.40	0.49	1.0000
Gasoline	0.9180	0.42	0.50	0.41	0.49	1.0000
Orange	1.0000	0.37	0.48	0.37	0.48	1.0000

Garlic	1.0000	0.56	0.50	0.56	0.50	1.0000
Banana	1.0000	0.29	0.46	0.30	0.46	1.0000
Mint	1.0000	0.43	0.50	0.43	0.50	1.0000

Supplementary Table 1: Chi-squared tests comparing correct identification rates between PD hyposmia (n=194) and non-PD hyposmia (n=194), per individual UPSIT item. Calculated p-values are subsequently adjusted for multiple comparisons. Red shading indicates a statistically significant difference ($p<0.05$). PD = Parkinson's disease; SD = standard deviation; UPSIT = University of Pennsylvania Smell Identification Test.

Feature set	ML model	Accuracy	Specificity	Sensitivity	Precision	F1-score
Response_SA	XGBoost	0.68	0.70	0.82	0.69	0.68
Response_SA	Gradient Boost	0.68	0.72	0.80	0.69	0.66
Response	Gradient Boost	0.68	0.74	0.79	0.70	0.66
Response	XGBoost	0.67	0.68	0.82	0.68	0.67
Response	Extra Trees	0.64	0.61	0.81	0.63	0.64
Response_SA	Extra Trees	0.63	0.63	0.80	0.63	0.63
Response	SVM	0.63	0.61	0.80	0.63	0.63
Response_SA	SVM	0.62	0.61	0.80	0.62	0.62
Response	Random Forest	0.61	0.59	0.80	0.61	0.62
Response_SA	Random Forest	0.61	0.61	0.78	0.61	0.61
Response	Ridge Regression	0.60	0.65	0.74	0.61	0.57
Response_SA	Ridge Regression	0.58	0.65	0.72	0.60	0.56
Correct_SA	Random Forest	0.56	0.61	0.72	0.57	0.54
Correct	Ridge Regression	0.56	0.60	0.73	0.57	0.55
Correct_SA	Ridge Regression	0.56	0.57	0.74	0.56	0.56
Correct_SA	Gradient Boost	0.55	0.53	0.75	0.55	0.56
Response	Decision Tree	0.55	0.52	0.76	0.55	0.56
Correct	Gradient Boost	0.54	0.54	0.74	0.54	0.54
Correct_SA	Extra Trees	0.54	0.56	0.72	0.54	0.53
Response_SA	Decision Tree	0.54	0.52	0.74	0.54	0.55
Correct	Random Forest	0.54	0.62	0.68	0.55	0.50
Response_SA	KNN	0.54	0.60	0.69	0.54	0.50
Correct	Extra Trees	0.54	0.56	0.71	0.54	0.52
Correct_SA	XGBoost	0.53	0.54	0.72	0.53	0.53
Correct_SA	KNN	0.53	0.56	0.71	0.53	0.52
Correct	Decision Tree	0.53	0.57	0.70	0.53	0.51

Response	KNN	0.52	0.51	0.73	0.52	0.53
Correct_SA	Decision Tree	0.52	0.51	0.73	0.52	0.52
Correct	SVM	0.52	0.52	0.72	0.52	0.52
Correct_SA	SVM	0.52	0.52	0.71	0.52	0.51
Correct	KNN	0.52	0.44	0.77	0.51	0.55
Correct	XGBoost	0.50	0.48	0.72	0.50	0.51

Supplementary Table 2: Performance analysis of ML approaches predicting PD based on UPSIT responses. Models were trained on a discovery cohort of hyposmic participants (194 PD hyposmia, 194 non-PD hyposmia) using four different combinations of features. Metrics were computed using leave-one-out cross-validation. KNN = K-Nearest Neighbours; ML = Machine Learning; SA = inclusion of sex and age; SVM = Support Vector Machine; UPSIT = University of Pennsylvania Smell Identification Test.

Feature set	ML model	Accuracy	Specificity	Sensitivity	Precision	F1-score
Response	XGBoost	0.68	0.67	0.83	0.68	0.68
Response	Gradient Boost	0.66	0.63	0.82	0.65	0.66
Response	Decision Tree	0.65	0.66	0.79	0.65	0.64
Response	SVM	0.63	0.61	0.81	0.63	0.64
Response_SA	XGBoost	0.63	0.58	0.82	0.62	0.65
Response_SA	SVM	0.63	0.61	0.80	0.62	0.63
Correct_SA	Random Forest	0.62	0.67	0.75	0.63	0.59
Response_SA	Ridge Regression	0.62	0.61	0.79	0.61	0.62
Response	Ridge Regression	0.61	0.61	0.78	0.61	0.61
Response	Extra Trees	0.61	0.61	0.78	0.61	0.61
Correct_SA	Gradient Boost	0.61	0.62	0.77	0.61	0.61
Correct	Gradient Boost	0.61	0.60	0.78	0.60	0.61
Response_SA	Gradient Boost	0.60	0.59	0.78	0.60	0.60
Correct	Random Forest	0.60	0.62	0.76	0.60	0.58
Response	Random Forest	0.60	0.58	0.78	0.59	0.60
Correct_SA	Decision Tree	0.60	0.60	0.77	0.60	0.59
Response_SA	Random Forest	0.60	0.58	0.78	0.59	0.60
Correct	Ridge Regression	0.59	0.61	0.76	0.59	0.58
Correct_SA	Ridge Regression	0.59	0.61	0.76	0.59	0.58
Response_SA	Decision Tree	0.59	0.60	0.76	0.59	0.59
Correct_SA	Extra Trees	0.58	0.56	0.77	0.58	0.59

Correct	XGBoost	0.58	0.58	0.76	0.58	0.57
Correct_SA	KNN	0.58	0.60	0.74	0.58	0.56
Response_SA	Extra Trees	0.58	0.60	0.74	0.58	0.56
Correct_SA	XGBoost	0.57	0.63	0.71	0.58	0.54
Correct	Extra Trees	0.57	0.60	0.73	0.57	0.55
Response	KNN	0.57	0.53	0.77	0.56	0.58
Correct_SA	SVM	0.57	0.56	0.76	0.56	0.57
Response_SA	KNN	0.57	0.66	0.69	0.58	0.52
Correct	SVM	0.56	0.55	0.76	0.56	0.56
Correct	Decision Tree	0.56	0.56	0.75	0.56	0.56
Correct	KNN	0.54	0.44	0.79	0.53	0.58

Supplementary Table 3: Performance analysis of ML models predicting PD based on UPSIT responses. Models were trained on a discovery cohort of hyposmic participants with no missing UPSIT data (125 PD hyposmia, 125 non-PD hyposmia) using four different combinations of features. Metrics were computed using leave-one-out cross-validation. KNN = K-Nearest Neighbours; ML = Machine Learning; SA = inclusion of sex and age; SVM = Support Vector Machine; UPSIT = University of Pennsylvania Smell Identification Test.

Feature set	ML model	Accuracy	Specificity	Sensitivity	Precision	F1-score
Response	Extra Trees	0.86	0.85	0.93	0.85	0.86
Response	Random Forest	0.85	0.85	0.93	0.85	0.85
Response_SA	Extra Trees	0.85	0.88	0.91	0.87	0.85
Response_SA	Random Forest	0.85	0.84	0.93	0.84	0.85
Response_SA	SVC	0.85	0.84	0.93	0.84	0.85
Response	SVC	0.85	0.86	0.91	0.86	0.85
Response	Gradient Boost	0.84	0.86	0.90	0.86	0.84
Correct	SVC	0.84	0.87	0.90	0.86	0.84
Response_SA	Gradient Boost	0.84	0.85	0.91	0.85	0.84
Correct_SA	SVC	0.84	0.86	0.91	0.85	0.84
Response	XGB	0.84	0.85	0.91	0.84	0.84
Response_SA	XGB	0.84	0.84	0.91	0.84	0.83
Correct_SA	Random Forest	0.83	0.82	0.91	0.82	0.83
Correct_SA	Ridge	0.82	0.81	0.91	0.81	0.82
Correct_SA	Extra Trees	0.82	0.83	0.90	0.83	0.82
Response	Ridge	0.82	0.83	0.90	0.82	0.82

Correct	Random Forest	0.82	0.88	0.87	0.86	0.81
Correct_SA	Gradient Boost	0.82	0.80	0.91	0.81	0.82
Response_SA	Ridge	0.81	0.87	0.87	0.85	0.80
Correct	Ridge	0.81	0.82	0.89	0.81	0.81
Correct	XGB	0.81	0.89	0.85	0.86	0.79
Correct	Gradient Boost	0.81	0.89	0.85	0.87	0.79
Correct	Extra Trees	0.80	0.80	0.89	0.80	0.80
Correct_SA	XGB	0.79	0.83	0.87	0.81	0.79
Correct_SA	KNN	0.79	0.80	0.89	0.80	0.79
Correct	KNN	0.79	0.82	0.87	0.80	0.78
Response	Decision Tree	0.77	0.89	0.81	0.86	0.74
Response_SA	Decision Tree	0.75	0.78	0.85	0.77	0.75
Correct	Decision Tree	0.70	0.73	0.82	0.71	0.70
Correct_SA	Decision Tree	0.70	0.89	0.72	0.82	0.64
Response	KNN	0.68	0.95	0.65	0.89	0.57
Response_SA	KNN	0.68	0.70	0.82	0.69	0.68

Supplementary Table 4: Performance analysis of ML models predicting PD based on UPSIT responses. Models were trained on data from 291 PD and 291 non-PD participants, where hyposmia status was disregarded during participant selection. Metrics were computed using leave-one-out cross-validation, using four different combinations of features. KNN = K-Nearest Neighbours; ML = Machine Learning; SA = inclusion of sex and age; SVM = Support Vector Machine; UPSIT = University of Pennsylvania Smell Identification Test.

Feature set	ML model	Accuracy	Specificity	Sensitivity	Precision	F1-score
Response	Random Forest	0.86	0.85	0.94	0.86	0.87
Response_SA	Extra Trees	0.86	0.83	0.95	0.85	0.87
Response_SA	Random Forest	0.86	0.83	0.94	0.85	0.86
Response	Extra Trees	0.83	0.81	0.92	0.82	0.84

Supplementary Table 5: Performance of the four best-performing approaches in the discovery cohort, using all UPSIT response features, with and without age and sex features, when applied to an independent validation cohort of 73 PD and 73 non-PD participants. Hyposmia status was disregarded during participant selection. ML = Machine Learning; SA = inclusion of sex and age; UPSIT = University of Pennsylvania Smell Identification Test.

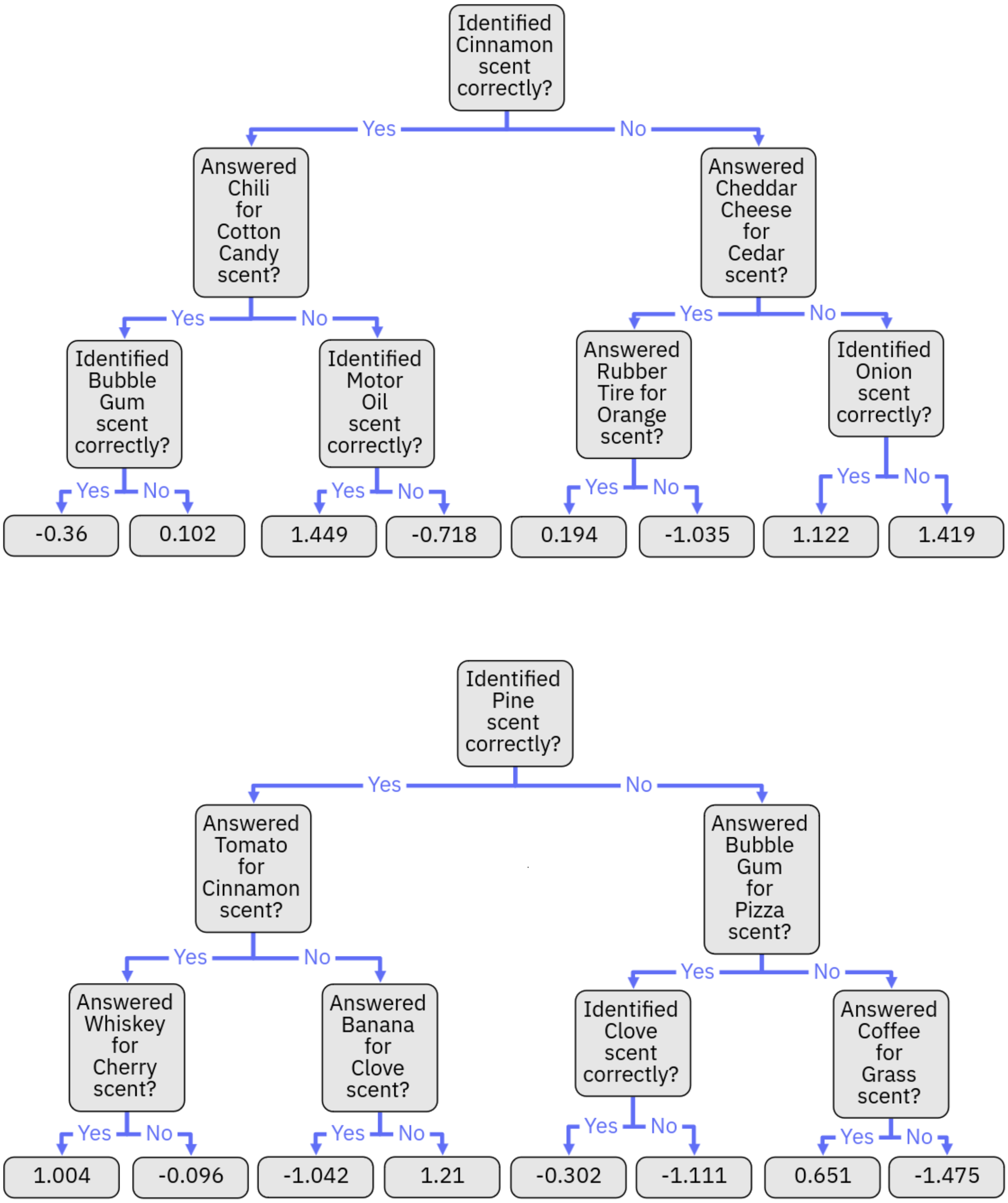

Supplementary Figure 1: Two illustrative examples of the 100 decision trees used by the trained Gradient Boost model with the Response_SA feature set. Overall model prediction is determined by the sum of the values at the end of each tree. A positive or negative final value indicates the prediction of PD or non-PD, respectively. Due to the large number of trees, direct analysis is challenging. Tools such as SHapley Additive exPlanations (SHAP) framework are used to summarise and highlight the most impactful response features (see Figure 3).
